# Field detection and predicted evolution of spinosad resistance in *Ceratitis capitata*

**DOI:** 10.1002/ps.5919

**Published:** 2020-06-04

**Authors:** Ana Guillem‐Amat, Lucas Sánchez, Elena López‐Errasquín, Enric Ureña, Pedro Hernández‐Crespo, Félix Ortego

**Affiliations:** ^1^ Departamento de Biotecnología Microbiana y de Plantas Centro de Investigaciones Biológicas Margaritas Salas Madrid Spain; ^2^ Departamento de Biología Celular y Molecular Centro de Investigaciones Biológicas Margaritas Salas Madrid Spain

**Keywords:** medfly, insecticide, nAChR, evolutionary model, resistance management

## Abstract

**BACKGROUND:**

The sustainable control of the Mediterranean fruit fly, *Ceratitis capitata* (Wiedemann), is compromised by the development of resistance to malathion and lambda‐cyhalothrin in Spanish field populations. At present, field populations remain susceptible to spinosad. However, the resistant strain JW‐100s has been obtained under laboratory selection with spinosad, and resistance has been associated with the presence of different mutations causing truncated transcripts of the α6 subunit of the nicotinic acetylcholine receptor (nAChRα6).

**RESULTS:**

An F1 screen assay followed by the molecular characterization of surviving flies has been used to search for spinosad‐resistant alleles in field populations. Two different resistant alleles giving rise to truncated isoforms of *Ccα6* have been identified, which corresponds to an estimated allelic frequency of at least 0.0023–0.0046. The fitness values of the resistant nAChRα6 alleles found in the laboratory strain JW‐100s were estimated to be 0.4 for *RR* and 0.2 for *SR*. Mathematical modelling predicted that spinosad‐resistant alleles will rapidly decline over time in field populations if their fitness cost was the same as estimated for laboratory‐resistant alleles. However, they are predicted to increase in the field if their fitness cost is lower and resistance management strategies are not implemented.

**CONCLUSION:**

Spinosad‐resistant alleles have been detected in field populations for the first time. Our modelling simulations indicate that the best option to delay the appearance of spinosad resistance would be its rotation with other insecticides without cross‐resistance. The integrated F1 screen/molecular genetic analysis presented here can be used for future monitoring studies. © 2020 The Authors. *Pest Management Science* published by John Wiley & Sons Ltd on behalf of Society of Chemical Industry.

## Graphical abstract

Spinosad‐resistant alleles have been found in field populations of *Ceratitis capitata* for the first time. Evolutionary modelling predicted that resistance management strategies will delay the evolution of resistance.

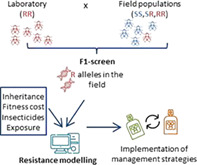

## INTRODUCTION

*Ceratitis capitata* (Wiedemann) (Diptera: Tephritidae), also known as the Mediterranean fruit fly (medfly), is considered one of the main insect pests for fruits due to the significant losses it causes to agriculture. In Spain, control practices against medfly rely on the use of insecticides, which is combined with the sterile insect technique (SIT) in some areas. Malathion was the most widely used insecticide in the 1990s and 2000s until its withdrawal from use in the European Union in 2009. Currently, applications of insecticides in citrus crops mainly consist of spinosad and lambda‐cyhalothrin as bait sprays, and deltamethrin in lure and kill traps.^1^ However, the sustainability of this control strategy is threatened by the development of insecticide resistance,^2^ which has already been reported for malathion^3^, ^4^ and lambda‐cyhalothrin.^5^ Monitoring of Spanish field populations performed in previous years found that all the analyzed populations were highly susceptible to spinosad.^6^ However, an extremely highly resistant strain (JW‐100s, about 2000‐fold) has been obtained by laboratory selection from a field‐derived population.^6^ In this context, the implementation of insecticide resistance management (IRM) strategies is required for both the improvement of medfly control programs and the sustainability of available insecticides.^7^, ^8^ Key components of an effective IRM strategy are the early detection of resistance, based on the use of robust and fast monitoring tools, and the reduction of selection pressure directed towards a particular insecticide, which favors the increase of resistant alleles in the population.

Spinosad is a natural product derived from the soil actinomycete *Saccharopolyspora spinosa*, which exerts a neurotoxic action by targeting the α6 subunit of the nicotinic acetylcholine receptor (nAChR).^9^, ^10^ The *α6* gene is highly conserved among insects, but can generate a diversity of α6 subunit proteins through post‐transcriptional modifications such as alternative splicing and A‐to‐I RNA editing.^11^ Spinosad resistance has been reported in field populations of several pest species,^12^, ^13^ including the tephritid flies *Bactrocera zonata* (Saunders)*,* ^14^ *B. oleae* (Rossi)^15^ and *B. cucurbitae* (Coquillett).^16^ Target‐site resistance has been described as the main cause of spinosad resistance, though there is also evidence of metabolic resistance mediated by P450 and esterases.^13^ Several studies have shown that alterations in the α6 subunit gene of the nAChR, such as mis‐splicings, indels or point mutations that generate premature stop codons or amino acid changes, result in resistance to spinosad.^10^, ^17^, ^18^, ^19^ It has been demonstrated that the knock‐out of α6 in *Drosophila melanogaster* (Meigen) does not cause lethality.^17^ Thus, it has been hypothesized that any mutation causing a loss of α6 function could confer resistance to spinosad, which makes resistance easier than if a specific mutation was required.^10^

In medfly, the *Ccα6* has shown a high plasticity that leads to the development of several alleles carrying different mutations associated with spinosad resistance in the laboratory‐selected strain JW‐100s.^6^ The participation of mutation Q68* in exon 3a in the resistant phenotype was functionally validated by ectopic gene expression in *D. melanogaster* using the GAL4 > UAS system. In addition, the generation of two isolines homozygous for each one of the alleles *Ccα6* ^3aQ68*Δ3b‐4^ (carrying Q68* and a deletion of exons 3b and 4) and *Ccα6* ^3aQ68*‐K352*^ (carrying Q68* and K352* in exon 10) allowed the determination that both alleles conferred the same level of resistance.^20^ Knowledge about the mutations conferring resistance have permitted the design of molecular markers for spinosad resistance in medfly, enabling the detection of the specific mutations Q68* and K352* observed in the JW‐100s strain.^6^ However, these or other mutations could appear in the field, so a broader strategy that allows searching for different mutations is required for spinosad resistance monitoring in field populations.

Theoretical evolutionary models are a relevant tool for resistance management, as they could contribute to decision making in IRM. They are based on empirical data^21^ and help to understand how resistant alleles can evolve in a population depending on biological/ecological (e.g. generation time, number of generations, migration rates), genetic (e.g. frequency and inheritance of resistance alleles, fitness costs of resistant individuals) and operational (e.g. timing, dose and formulation of insecticides used, cross‐resistance between insecticides) factors.^7^ This type of model is commonly implemented in mosquitos in the fight against malaria as they are useful to predict, manage and prevent resistance evolution.^22^, ^23^ In the case of medfly, some of the information needed for this purpose is already available. Our previous work determined that the inheritance of spinosad resistance in JW‐100s was autosomic and completely recessive.^6^ In addition, we showed that spinosad resistance imposes a fitness cost to resistant individuals compared to susceptible ones, since the resistant *Ccα6* ^3aQ68*‐K352*^ and *Ccα6* ^3aQ68*Δ3b‐4^ alleles were not stable when in competition with the wild‐type allele.^20^ Remarkably, we also demonstrated that spinosad resistance could have an impact on the behavior of this species, affecting the ability of males homozygous for the *Ccα6* ^3aQ68*Δ3b‐4^ allele to detect the parapheromone trimedlure and to mate in competition experiments.^20^ However, the fitness values of these resistant genotypes have not been estimated, and the frequency of resistance alleles in the field is unknown.

In the present study we have (i) developed and implemented a methodology for the detection of resistant alleles in field populations, (ii) reanalyzed the data from the study of the stability of the two resistant genotypes found in the JW‐100s strain^20^ to estimate their fitness value and (iii) predicted how resistance would evolve in the field based on evolutionary models.

## MATERIALS AND METHODS

### Laboratory strains and field populations of *Ceratitis capitata*

The susceptible laboratory strain of *C. capitata*, C, originally set up in 2001 from non‐treated experimental fields at the Instituto Valenciano de Investigaciones Agrarias (IVIA, Valencia, Spain), has been reared in the laboratory without refreshing the colony with wild individuals and without exposure to insecticides. The spinosad‐resistant strain JW‐100s, derived from field individuals collected in Xàbia in 2007, has been maintained under regular selection in the laboratory, as previously reported.^6^ At the time the experiments were performed, the strain was constituted by individuals homozygous for the *Ccα6* ^3aQ68*‐K352*^ allele and heterozygous for the *Ccα6* ^3aQ68*‐K352*^ and *Ccα6* ^3aQ68*Δ3b‐4^ alleles.^6^, ^20^ The isoline Q68*‐K352* is homozygous for the allele *Ccα6* ^3aQ68*‐K352*^ and was obtained by isolating it from JW‐100s strain.^20^

Field populations were collected from orchards sited in different localities of East Spain, in the Mediterranean area, during the years 2016–2018 (Table 1). Fruit punctured by *C. capitata* was taken from the field to the laboratory, placed in ventilated plastic boxes (15 × 21 × 28 cm) and kept at controlled conditions [photoperiod of 16:8 h light:dark and a temperature of 26 ± 2 °C (light) and 22 ± 2 °C (dark)]. New pupae were harvested every 2–3 days and individualized, and adults were sexed immediately after emergence. Males were kept in ventilated boxes (12 cm in diameter and 5 cm height) and provided with water and rearing diet (4:1 sugar: yeast) *ad libitum* in an environmentally controlled chamber (Sanyo MLR‐350‐H, Sanyo, Japan) at 25 ± 1 °C and 16 h light and 8 h dark photoperiod (standard conditions).

**Table 1:** Field populations of *C. capitata*

Population	Year	Host	Field treatment^a^
Algarrobo Costa (Málaga)	2016	Cherimoya	Non‐treated in recent years (experimental field)
Oliva (València)	2016	Citrus	Spinosad in 2016
Sanet i Negrals (Alacant)	2017	Citrus	Spinosad in 2017 (5×)
Onda (Castelló)	2017	Citrus	Spinosad in 2017 (3×)
Alcalà de Xivert (Castelló)	2017	Citrus	Non‐treated / Spinosad in 2017 (3×)^b^
Puçol (València)	2018	Citrus	Spinosad in 2018 (3×)

^a^ The number in parentheses is the number of field applications of bait formulation with spinosad (by ground or aerial treatment) per year.

^b^ This population came from two different fields, one that had no insecticide treatment and another treated with spinosad.

### F1 screen assays

Groups of males from field populations were crossed with virgin females from the laboratory Q68*‐K352* isoline or JW‐100s strain, both resistant to spinosad, in the proportion 1:1 male:female. Crosses were performed in methacrylate cages (20 × 20 × 20 cm), with a cloth mesh in one of their sides, containing water and rearing diet *ad libitum*. Crosses were performed only in one direction because females from laboratory strains can lay eggs through the cloth mesh, while wild females need to be adapted. All the resultant F1 eggs were spread over larvae rearing diet [stock prepared by mixing 300 g of sugar, 11.2 g of *N*‐propil‐*p*‐hydroxibenzoate (Sigma‐Aldrich, St. Louis, MO, USA), 11.2 g of methyl‐4‐hydroxibenzoate (Sigma‐Aldrich, St. Louis, MO, USA), 10 g of benzoic acid (Merck Darmstadt, Germany), 145.2 g of dry beer yeast (Difco Laboratories, Erembodegem, Belgium), 1000 g of wheat bran and 2400 mL of distilled water] with 5 ppm of spinosad (Dow AgroSciences 88% p/p, Indianapolis, IN, USA), covered with aluminum foil and kept at standard conditions. The development was checked frequently to isolate and provide with water and rearing diet those individuals that reached the adult stage.

### Sequencing of *Ccα6*

The gene of the α6 subunit of the nicotinic acetylcholine receptor (*Ccα6*) was studied in surviving F1 screen assay flies. RNA extraction and complementary DNA (cDNA) synthesis was performed on the heads of adult flies when they were in a good state and on whole bodies when flies were amorphous or the head was not in good condition. Total RNA extraction was performed with TRIzol® Reagent (Life Technologies, Van Allen Way, Carlsbad, CA, USA) according to the manufacturer's instructions. Quantification of RNA was performed using Nanodrop ND‐1000 spectrophotometer (Thermo Fisher Scientific, Waltham, MA, USA). Reverse transcription to cDNA used 2 μg of RNA as a template and a commercial kit (Thermo Scientific). Before following the kit indications, RNA was incubated with RQ1 DNase (Promega, Madison, WI, USA) at 37 °C for 35 min to remove gDNA, and the reaction was stopped by incubation with the RQ1 DNase Stop solution at 70 °C for 10 min.

PCR was performed in a volume of 10 μL using 0.4 μm of oligonucleotides FnACh6ex1 (5′‐CAACGGAAGCTGAAATCTAAGGAC) and RnACh6ex12nest (5′‐TCGCTGTTTGCCGTGTTGATCTTT), 2 U of iTaq™ DNA Polymerase (Biorad, CA, USA), 5x HF Buffer, 0.2 mm dNTPs (Thermo Fisher Scientific, Austin, TX, USA) and 5 μL of the corresponding cDNA diluted 2‐fold as template. PCR conditions were as follows: an initial denaturation step at 98 °C for 30 s; 40 cycles of 98 °C for 10 s, 60 °C for 30 s and 72 °C for 1.5 min; and a final step of 72 °C for 7 min for full extension. Nested‐PCR was performed following the same procedure, using 5 μL of the first PCR product diluted 10‐fold as a template and oligonucleotides FnACh6ex2–76 (5′‐CGTCTACTTAACCACCTATTATCC), RnACh6ex5 (5′‐CGTGCCATCGAATCCCTCATCC), RnACh6ex11–1382 (5′‐CTATCCACAACCATTGCCGCAAAC) and RnACh6ex12‐nest (5′‐TCGCTGTTTGCCGTGTTGATCTTT). PCR products were analyzed by electrophoresis on 1.5% agarose gel (Agarose D2, Conda Pronadisa) and purified (QIAquick PCR Purification Kit, Qiagen, Germany). When needed, bands were cut from the gel and eluted with centrifugal filter units (Merck Millipore, Darmstadt, Germany). PCR products were sequenced (Secugen, Madrid, Spain) and analyzed using Genious 11.0.5 (https://www.geneious.com).

### Evolutionary model of spinosad resistance

A model to predict the evolution of spinosad resistance was established. It was assumed to be a panmictic population with discrete generations, and it was considered that the effective population size is large enough to rule out genetic drift effects. Several parameters with different values were tested in the simulation (Table 2).

**Table 2:** Parameter values used in the evolutionary model for spinosad resistance in *C. capitata*

Parameter	Definition		
*w*_*XY*_	Fitness cost of genotype *XY* = {(*SS*), (*RR*), (*SR*)}; 0 ≤ *w*_*XY*_ ≤ 1		
*s*(*s*)_*XY*_	Sensitivity to spinosad (*s*) of genotype *XY*; 0 ≤ *s*(*i*)_*XY*_ ≤ 1		
*s*(*i*)_*XY*_	Sensitivity to insecticide (*i*) with no cross‐resistance to spinosad of genotype *XY*; 0 ≤ *s*(*i*)_*XY*_ ≤ 1		
*e*(*i*)	Exposition to insecticide (*i*), understood as the percentage of insects in the population contacting the insecticide, *e*(*i*) = {(0.2), (0.5), (0.8), (0.95)}		
IF_*XY*_	Initial frequency of genotype *XY* = {(*SS*), (*RR*), (*SR*)} used to calculate the predicted rate of resistance evolution		
**Fitness cost of genotype *XY* (*w***_***XY***_ **) (see Fig.** **1** **)**			
**Scenario (SC)**	***w_SS_***	***w_RR_***	***w_SR_***
SC0	0	0.4	0.2
SC1	0	0.13	0.07
SC2	0	0.04	0.02
SC3	0	0.01	0.007
SC4	0	0.005	0.002
**Expected mortality of the genotypes to field spinosad concentration (260 ppm)**			
*s*(*s*)_*SS*_	*s*(*s*)_*RR*_	*s*(*s*)_*SR*_	
1	0	1	
**Expected mortality of the genotypes with an insecticide with no‐cross resistance to spinosad**			
*s*(*i*)_*SS*_	*s*(*i*)_*RR*_	*s*(*i*)_*SR*_	
1	1	1	
**Initial frequencies IF1; F(*R*) = 0.0023 (2 resistant alleles/880)**			
F(*SS*)	F(*RR*)	F(*SR*)	
0.9955	0.000005	0.0045	
**Initial frequencies IF2; F(*R*) = 0.0046 (4 resistant alleles/880)**			
F(*SS*)	F(*RR*)	F(*SR*)	
0.9909	0.00002	0.0091	

The simulation was developed to detect the evolution of three different genotypes that could compose a field population: *SS, RR* and *SR*, where *S* is a susceptible allele and *R* is a resistant allele of the *Ccα6* gene, carrying any mutation. The initial frequency of each genotype was calculated considering the frequency of appearance of resistant alleles, taking this information from the F1 screen performed with populations collected during the years 2016–2018 (see section 3.1). Two situations were simulated: considering that both field individuals carrying spinosad resistance alleles (from 440 tested) were heterozygous for the resistant allele (IF1 = 0.0023) or that they were homozygous (IF2 = 0.0046). The case corresponding to one heterozygous individual and one homozygous individual was not considered.

To calculate the fitness of each genotype (equivalent to 1‐*w*_*XY*_, where *w*_*XY*_ is the fitness cost or selection coefficient of genotype *XY*), it was considered that, by definition, the wild‐type (*SS*) had no fitness cost (*w*_*SS*_ = 0). To simplify the understanding of the evolutionary model, from here on we will talk about fitness cost instead of selection coefficient. For the remaining genotypes, we simulated the evolution of resistance assuming that the fitness cost of all field‐resistant alleles would be the same as that estimated for the laboratory‐resistant alleles [scenario (SC) 0 = 0.4 for *RR* and 0.2 for *SR*, see section 3.2], as well as scenarios where lower fitness costs were considered (SC1 = 0.13 for *RR* and 0.07 for *SR*; SC2 = 0.04 for *RR* and 0.02 for *SR*; SC3 = 0.01 for *RR* and 0.007 for *SR*; and SC4 = 0.005 for *RR* and 0.002 for *SR*).

Different scenarios of insecticide treatments strategies were tested that combined none, one or two insecticide applications during three consecutive generations of *C. capitata*, using spinosad only or in combination with a second insecticide without cross‐resistance to spinosad. The concentration of spinosad was selected according to that established for field treatments (260 ppm in bait sprays). Considering that spinosad resistance is inherited as a recessive trait,^6^ the sensitivity to spinosad was defined as *s*(*i*)_*SS*_ = *s*(*i*)_*SR*_ = 1, as both heterozygous and wild‐type homozygous would die when exposed to a field dose of spinosad. As we showed in previous works,^20^ this concentration has no effect on resistant homozygous individuals, for which *s*(*i*)_*RR*_ = 0. The expected mortality for all genotypes when exposed to an insecticide with no cross‐resistance was considered to be *s*(*i*)_*XY*_ = 1.

As the insecticide treatment is considered to reach only part of the population, four levels of insecticide exposure (*e*(*i*)) were considered: 20%, 50%, 80% and 95%. The population was therefore divided into two subpopulations depending on whether or not the insect contacted with the insecticide. Note that the putative migration is implicit in the parameter *e*(*i*). The general equation for calculating the relative genotype frequency through generations was:

$$F_{n+1}\left(\mathrm{XY}\right)=\frac{\begin{array}{c} \left\{\left[\sum \left[F_{n}\left(\mathrm{XY}\right)\times F_{n}\left(\mathrm{XY}\right)\times F\left(\mathrm{XY}\right)\right]\;\right]\times \left(1-w_{\mathrm{XY}}\right)\times \left(1-s{\left(s\right)}_{\mathrm{XY}}\right)\times \left(1-s{\left(i\right)}_{\mathrm{XY}}\right)\times e\left(i\right)\right\}\\ +\\ \left\{\left[\sum \left[F_{n}\left(\mathrm{XY}\right)\times F_{n}\left(\mathrm{XY}\right)\times F\left(\mathrm{XY}\right)\right]\;\right]\times \left(1-w_{\mathrm{XY}}\right)\times \left(1-e\left(i\right)\right)\right\} \end{array}}{\left[\sum F_{n+1}\left(\mathrm{all}\;\mathrm{XY}\;\text{genotypes}\right)\right]}$$

Genotype (*XY*) = {(*SS*), (*RR*), (*SR*)}

$$F_{n+1}\left(\mathrm{XY}\right)=\text{frequency of genotype}\;\mathrm{XY}\;\mathrm{at}\;\text{generation}\;\left(n+1\right)$$

$$F_{n}\left(\mathrm{XY}\right)=\text{frequency of genotype}\;\mathrm{XY}\;\mathrm{at}\;\text{generation}\;\left(n\right)$$

$$\left[F_{n}\left(\mathrm{XY}\right)\times F_{n}\left(\mathrm{XY}\right)\right]=\text{frequency of}\;\mathrm{any}\;\text{cross between genotypes}\;\mathrm{XY}\;\text{and}\;\mathrm{XY}$$

$$F\left(\mathrm{XY}\right)=\text{frequency of genotype}\;\mathrm{XY}\;\text{produced}\;\mathrm{by}\;a\;\text{given cross}$$

The simulation covered 60 generations (10 years, since *C. capitata* usually has six generations per year in the area of study).

## RESULTS

### Search for resistant alleles in field populations of *C. capitata*

We developed an F1 screen assay followed by a molecular characterization of *Ccα6* in spinosad surviving flies to identify resistant alleles in the field. The F1 screen assay is based on crosses between field‐collected (of unknown genotype) and laboratory individuals of the resistant isoline Q68*‐K352*, homozygous for the resistant allele *Ccα6* ^3aQ68*‐K352*^, or from the resistant strain JW‐100s, composed of individuals homozygous for *Ccα6* ^3aQ68*‐K352*^ or heterozygous for *Ccα6* ^3aQ68*Δ3b‐4^ and *Ccα6* ^3aQ68*‐K352*^. Since spinosad resistance is completely recessive,^6^ only the F1 offspring with a resistant allele from the field‐collected parental and a resistant allele from the resistant isoline/strain will survive when exposed to a discriminatory concentration of spinosad.

From a total of 440 males screened, 13 F1 survivors to spinosad (pupae, pharates or adults) were obtained from three (Oliva, Algarrobo Costa and Puçol) of the six populations tested (Table 3). When we amplified by PCR and sequenced the *Ccα6* gene in all 13 individuals, we found mutations in the *Ccα6* gene corresponding to the field parental in two of them, an adult from Algarrobo Costa (K352*) and another from Puçol (deletion of exons 5–11). The two mutations were obtained from different F1 crosses, so they were necessarily inherited from two different field‐collected males. We did not find mutations corresponding to the field parental in the rest of survivors, probably because they were false positives that would have escaped the insecticide in the diet. However, we cannot discount that mutations in a gene different to *Ccα6* or alterations in non‐coding parts of this or other gene could be causing the resistance. Thus, we can conclude that spinosad‐resistant alleles are present in at least two field‐collected individuals (from 440 tested), which corresponds to an allelic frequency of 0.0023 if both were heterozygous for the resistant allele or 0.0046 if they were homozygous.

**Table 3:** F1 screen performed by crossing males from *C. capitata* field populations and females from the laboratory JW‐100s strain or Q68*‐K352* isoline, and detection of mutations in *Ccα6* in the F1 survivors

Population/strain	Year	Tested Males (*n*)	F1 survivors (*n*)^a^	Mutations at *Ccα6* ^b^ in the F1 survivors
Oliva (València)	2016	70	1 pharate + 2 pupae	None
Algarrobo Costa (Málaga)	2016	140	3 adults + 1 pupa	K352* in 1 adult
Sanet i Negrals (Alacant)	2017	25	0	None
Alcalà de Xivert (Castelló)	2017	25	0	None
Onda (Castelló)	2017	40	0	None
Puçol (València)	2018	140	4 adults + 2 pharates	Absence of exons 5–11 in 1 adult

^a^ Number of F1 survivors to a discriminatory dose of 5 ppm in larvae diet.

^b^ Mutations refer to the copy of the *Ccα6* gene corresponding to the ‘field’ parental. In all cases, mutations Q68* and K352* corresponding to the parental JW‐100 s strain or Q68*‐K352* isoline were found in the other copy of the *Ccα6* gene.

### Estimation of the fitness cost of resistant genotypes under laboratory conditions

**Figure 1:**
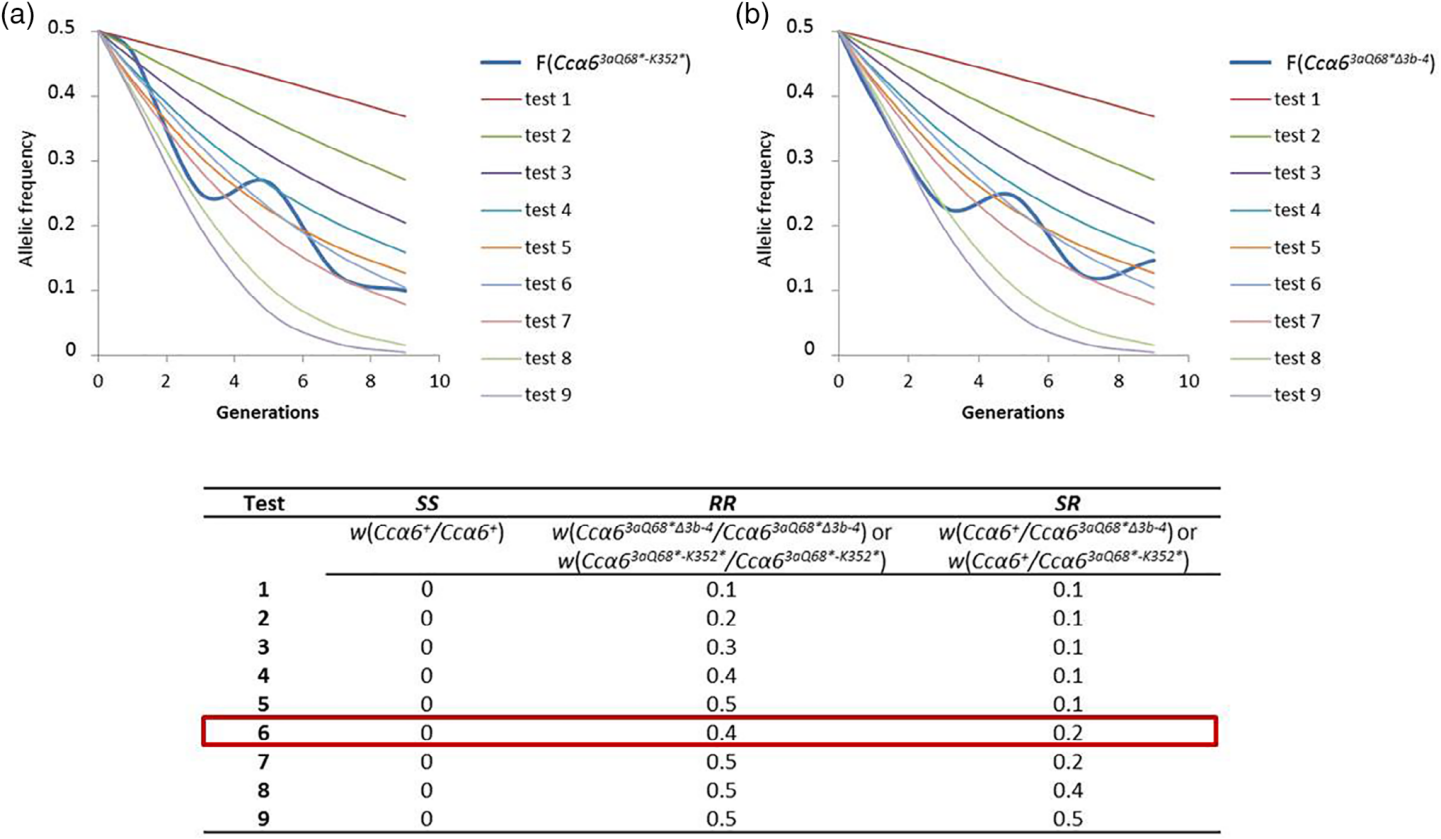
Estimation of the fitness cost associated with spinosad resistance in *C. capitata* under laboratory conditions. Experimental frequency (*F*) evolution of alleles *Ccα6* ^3aQ68*‐K352*^ (a) and *Ccα6* ^3aQ68*Δ3b‐4^ (b) obtained in a study of their stability when in competition with individuals carrying the wild‐type allele *Ccα6* ^*+*^.^20^ Different tests represent scenarios with different fitness cost values assigned to each genotype. The test with more intersections with the allelic frequency obtained from experimental data is considered the one that best adjusts to the evolution of resistance in the laboratory.

Trial and error modelling was performed to determine the combination of fitness cost values that better adjusted the expected evolution of the *R* allele (Fig. 1). The frequencies were obtained from a previous experimental laboratory study in which the stability of the spinosad‐resistant alleles *Ccα6* ^3aQ68*‐K352*^ and *Ccα6* ^3aQ68*Δ3b‐4^ was determined under laboratory conditions when in competition with individuals carrying the wild‐type allele *Ccα6* ^*+*^.^20^ We tested nine different combinations of fitness cost values and selected the one with more intersections with the allelic frequency obtained from experimental data. The combination of values was the same for both resistant alleles *Ccα6* ^3aQ68*‐K352*^ (Fig. 1(a)) and *Ccα6* ^3aQ68*Δ3b‐4^ (Fig. 1(b)), and resulted in 0.4 for *RR* and 0.2 for *SR* (test 6 in Fig. 1).

### Modelling of spinosad resistance evolution

An evolutionary model for spinosad resistance was established, using for the parameterization of the model our estimates on the inheritance,^6^ allelic frequency (see Section 3.1) and fitness costs (see Section 3.2) of resistant alleles, and the information available on the biology, ecology and control of medfly in citrus crops in the Comunitat Valenciana (East Spain, Mediterranean area), the largest producer of citrus fruits in Europe. Treatments with spinosad for medfly control are normally performed during generations G4–G6 (*C. capitata* usually has six generations per year in the area of study) from September to October/November due to the phenology of the crop. During this period, one to six bait spray treatments are applied, mainly ground treatments although aerial treatments can also be used in the case of a severe attack. They can be performed with spinosad as the only insecticide used, rotated with lambda‐cyhalothrin or in combination with lure‐and‐kill traps coated with deltamethrin. Medfly populations can also be treated with insecticides early in the year, coinciding with generations G2–G3, when it is attacking other host plants. Simulations of the evolution of the three possible genotypes (*SS, SR* and *RR*) under different scenarios were performed. The analysis consisted of calculating the frequencies of these genotypes over generations. The genic frequencies were then inferred from genotypic frequencies. To facilitate the interpretation of the figures, only the evolution of the susceptible *S* allele is shown (note that knowing the frequency of allele *S*, that of allele *R* is complementary as both frequencies equal 1).

**Figure 2:**
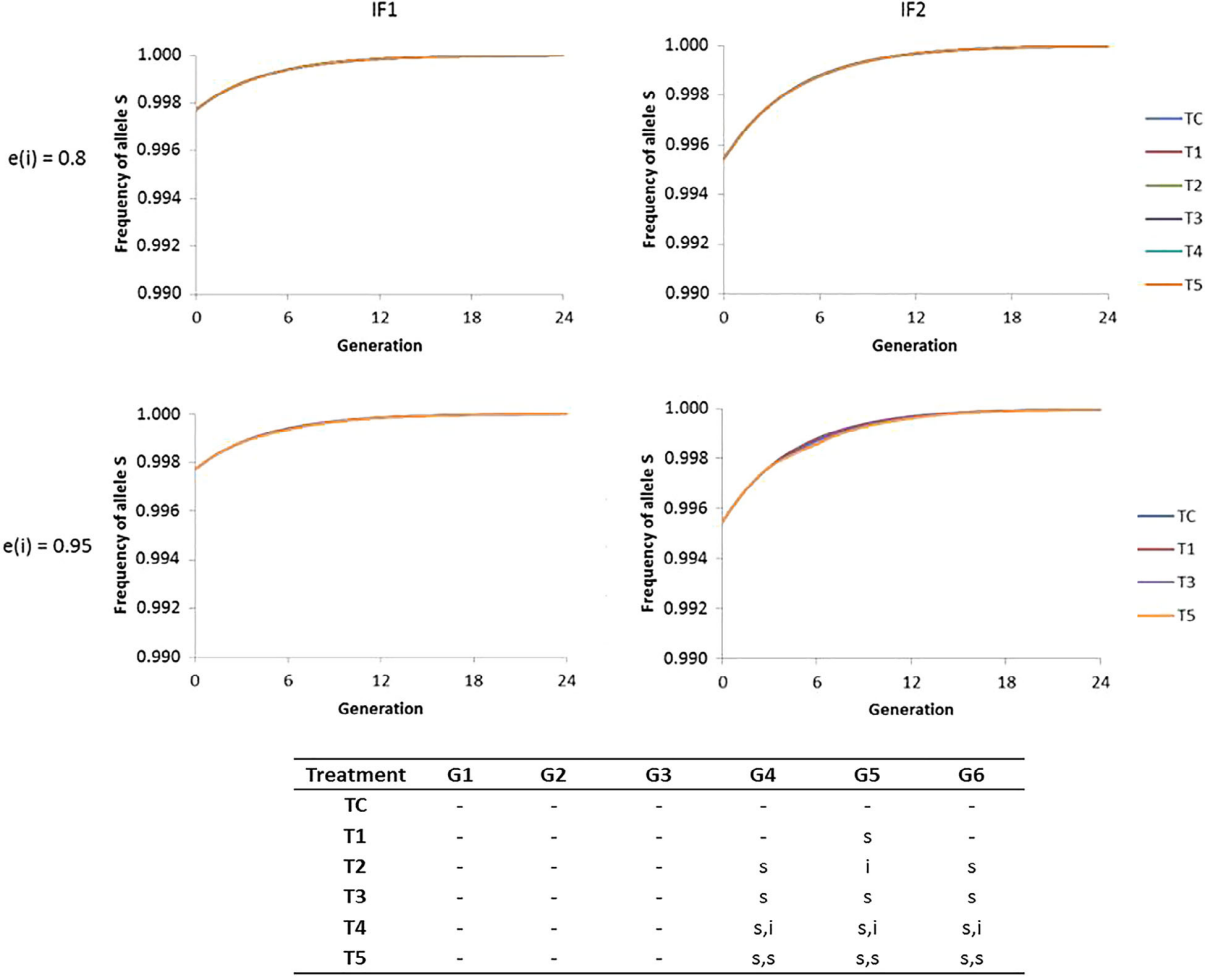
Predicted rate of the evolution of the spinosad susceptible allele (*S*) frequency in field populations of *C. capitata* (24 generations, six generations (G1–G6)/year) when using spinosad (s) and a second insecticide without cross resistance (i), under different resistance management strategies (T1–T5 and TC). Note that one treatment [(s) or (i)] or two treatments [(s,s) or (s,i)] can be performed per generation. The parameters used in the model are detailed in Table 2, with fitness cost values *w*_*RR*_ = 0.4 and *w*_*RS*_ = 0.2. Two levels of insecticide exposure *e(i)*, 80% and 95%, and two initial frequencies, corresponding to the presence of two [IF1; F(*S*) = 0.9977] or four [IF2; F(*S*) = 0.9955] resistant alleles (*R*) out of 880 analyzed, were considered.

We first tested the evolution of resistance assuming that the fitness cost of all field‐resistant alleles would be the same as that estimated for the laboratory‐resistant alleles *Ccα6* ^3aQ68*‐K352*^ and *Ccα6* ^3aQ68*Δ3b‐4^ under laboratory conditions (0.4 for *RR* and 0.2 for *SR*). The model predicted that the *S* allele will rapidly get fixed in the population and the *R* allele will be eliminated under all the different resistance management strategies analyzed (Figs 2 and S1). We observed this result for both initial frequencies of the resistant alleles considered (IF1 or IF2), and even in the scenarios with the highest exposure (*e*(*i*) = 0.95) and the biggest number of spinosad treatments per year. So, considering that spinosad resistance had a fitness cost in the wild equivalent to the one we found in the laboratory, resistant alleles would rapidly decline over time in field populations.

**Figure 3:**
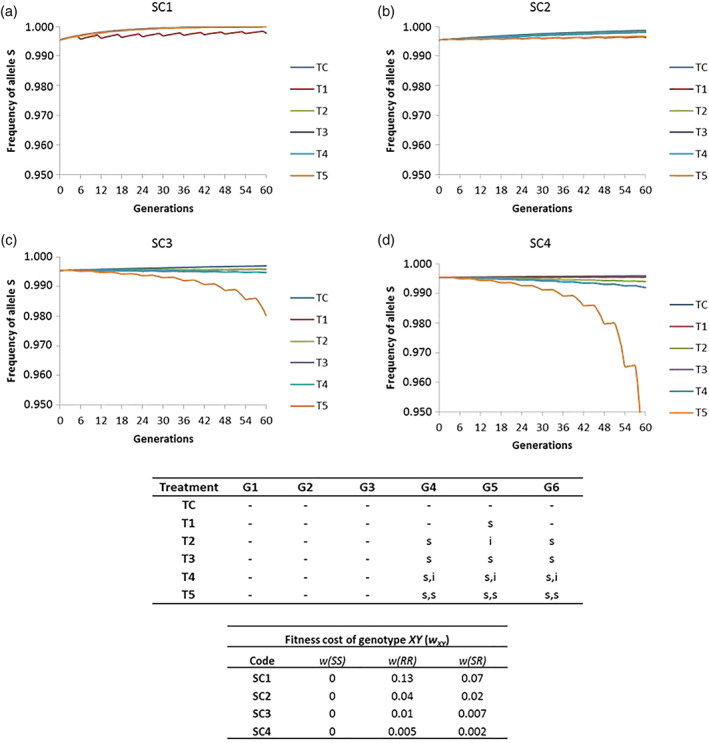
Predicted rate of the evolution of the spinosad susceptible allele (*S*) frequency in field populations of *C. capitata* (60 generations, six generations (G1–G6)/year) when using spinosad (s) and a second insecticide without cross resistance (i), under different resistance management strategies (T1–T5 and TC) and different values of fitness cost: (a) SC1, (b) SC2, (c) SC3 and (d) SC4. Note that one treatment [(s) or (i)] or two treatments [(s,s) or (s,i)] can be performed per generation. The parameters used in the model are detailed in Table 2. A level of insecticide exposure *e(i)* = 80% and an initial frequency corresponding to the presence of four [IF2; F(*S*) = 0.9955] resistant alleles (*R*) out of 880 analyzed were considered.

However, there is a chance that the fitness cost of spinosad resistance in the field is different to that observed in the laboratory. To explore this possibility, scenarios with different fitness costs were considered (Fig. 3). It was observed that the lower the fitness cost was for the resistant allele, the faster it increased in the population. This result was especially remarkable when considering an initial frequency IF2 F(*R*) = 0.0046 and high insecticide exposure (80%). Under these conditions, resistant alleles are predicted to increase in the population if fitness cost is reduced 40 times (SC3, *w*_*RR*_ = 0.01 and *w*_*SR*_ = 0.007) and six treatments with spinosad are applied per year (T5) (Fig. 3(c)). When fitness cost is reduced 80 times (SC4, *w*_*RR*_ = 0.005 and *w*_*SR*_ = 0.002), resistant alleles are expected to slightly increase with two (T2) and three (T3 and T4) treatments per year, and with six treatments per year (T5) they would reach 5% before 10 years (60 generations) (Fig. 3(d)). However, resistant alleles will eventually be eliminated if the fitness cost is only reduced three (Fig. 3(a)) or even 10 times (Fig. 3(b)). Altogether, our modelling results suggest that, if spinosad resistance evolved in the field is associated with a low fitness cost, resistant individuals would rise in the population if the treatments with this insecticide were abundant, while the reduction in the number of treatments of spinosad per year, or the rotation of this insecticide with another without cross‐resistance, would relax its selection pressure.

## DISCUSSION AND CONCLUSIONS

We have found spinosad‐resistant alleles in field populations of *C. capitata* for the first time. The early detection of resistant alleles in field populations, before insecticide efficacy is affected, is crucial for the implementation of resistance management strategies and requires effective monitoring systems.^7^, ^24^ Routine monitoring by bioassays remains the basis of most programs, but much attention is being paid to developing techniques that offer greater precision.^7^, ^25^ Taking advantage of the fact that (i) medfly strains and isolines with resistance to spinosad mediated by mutations in the *Ccα6* have been obtained by laboratory selection^6^, ^20^, (ii) spinosad resistance in the laboratory strain is completely recessive^6^ and (iii) different mutations in the *Ccα6* spinosad target site giving rise to truncated transcripts can lead to resistance in *C. capitata,* ^6^, ^20^ we developed an F1 screen assay to detect new *Ccα6*‐resistant alleles in field populations. It is based on the cross between field‐collected (of unknown genotype) and laboratory individuals of a resistant strain,^6^ followed by molecular characterization of *Ccα6* in spinosad surviving progeny. If a field individual carries spinosad‐resistant alleles in the *Ccα6* gene, the progeny will be resistant because they will inherit resistant alleles at the same locus from both parents. Interestingly, we were able to identify two new resistant alleles giving rise to truncated isoforms of *Ccα6*: one carrying the mutation K352* and the other producing transcripts that lacked exons 5–11, which could be due to a deletion or to a splicing alteration. The mutation K352* had previously been observed in the resistant JW‐100s strain, but in that strain it was always linked to the mutation Q68* in the *Ccα6* ^3aQ68*‐K352*^ allele.^6^ This is an example of the insects' nAChR inherent plasticity, through which evolution can select different alterations to overcome selection pressure.^26^ In addition, the F1 screen assay has allowed a first estimation of the allelic frequency of spinosad‐resistant alleles in Spanish field populations (0.0023–0.00446), which can be used for modelling the potential evolution of resistance under different treatment strategies. It should be noted that these allelic frequencies may be underestimating the real situation in the field, since field individuals carrying spinosad‐resistant alleles at different loci than the *Ccα6* gene will not be detected through our F1 screening because allelic complementation is expected to restore susceptibility.^27^ Nevertheless, the allelic frequencies estimated represent an approximation which can be used as a basal line for future monitoring studies.

Mathematical modelling predicted that if the fitness cost of spinosad‐resistant alleles detected in field medfly populations was the same as estimated for laboratory isolines, they will rapidly decline over time in field populations. We obtained this result for all the scenarios simulated and even in the more extreme case where spinosad was the only insecticide used, 95% of the population was exposed to the insecticide and six treatments were applied per year. A previous study determined that spinosad resistance entails a fitness cost, as observed for Q68* and Q68*‐K352* isolines, carrying the *Ccα6* ^3aQ68*Δ3b‐4^ and *Ccα6* ^3aQ68*‐K352*^ alleles, respectively.^20^ We have reanalyzed the data of that study using our evolutionary model and found that the estimated fitness cost values were the same for both resistant alleles under laboratory conditions (*w*_*RR*_ = 0.4 and *w*_SR_ = 0.2). To test if these values of fitness cost were compatible with the estimated frequency of resistant alleles in field populations, we assumed that the current frequency of resistant alleles was maintained by selection‐mutation equilibrium, where the net decrease in the resistant mutant alleles due to their fitness cost was compensated by the net increase in the amount of new resistant alleles generated by *de novo* mutations (Supporting Information Appendix S1). Our model indicates that under these conditions, the spinosad‐resistant *de novo* mutation rate would range from 1.4 × 10^−5^ to 8 × 10^−5^, which is within the usual range for resistant mutations in insects.^7^ However, care should be taken when extrapolating the fitness costs estimated for laboratory strains to field populations because their genetic backgrounds and environmental conditions are not alike.^28^, ^29^ Remarkably, our simulations showed that a considerable reduction in fitness cost should occur to result in an increase in resistant alleles in field populations. Nonetheless, there are several examples of absence of trade‐offs in field populations, which developed resistance as a result of selection of alleles with no fitness cost, selection of compensatory mutations that ameliorate the fitness of resistant genotypes or the replacement of resistant alleles by less costly ones.^28^, ^30^, ^31^ Indeed, it has been suggested that genetic variability in field populations can compensate to some extent for the fitness cost associated with some resistant genotypes.^28^

This work has allowed the detection of resistant alleles in field populations before a reduction of insecticide efficacy could be detected with bioassays.^6^ Attention should therefore be paid to avoiding an increase in their allelic frequency, especially in the present situation in which insecticide resistance to malathion^4^ and lambda‐cyhalothrin^5^ has already been reported in the same areas. Currently, the repertoire of effective insecticides against this pest is becoming very limited, which constraints Spanish farmers to use only one or a few effective insecticides and seriously compromises medfly control. In this context, a reinforcement of resistance management strategies is required. Our results indicate that the best option to delay the appearance of spinosad resistance is the rotation with available insecticides (lambda‐cyhalothrin or deltamethrin). Our simulation model showed that even in an extreme scenario with high exposure to spinosad (80%) and very low fitness cost (*w*_*RR*_ = 0.005 and *w*_*SR*_ = 0.002), the selection of resistant alleles could be delayed to avoid control failures if (i) a reduction in the number of spinosad treatments to a maximum of once per generation happened and/or (ii) spinosad was applied on rotation with a second insecticide without cross‐resistance. However, care should be taken in those fields where only spinosad is used year after year, as its overuse would favor the quick fixation of resistant alleles in small or isolated populations, as reported in other species.^32^ In addition, for a rational use of chemical products and a better management of resistance, the insecticide treatments should be harmonized with other control methods, such as SIT and cultural practices. Finally, the availability of molecular techniques for monitoring spinosad resistance alleles will allow detection of significant changes before widespread field failure, so that adjustments to the resistance management strategies can be made to ensure the sustainability of the insecticide.

## Supporting information


**Figure S1.** Predicted rate of the evolution of the spinosad susceptible allele (*S*) frequency in field populations of *Ceratitis capitata* (24 generations, six generations (G1–G6)/year) when using spinosad (s) and a second insecticide without cross resistance (i), under different resistance management strategies (T1–T5 and TC). Note that one treatment [(s) or (i)] or two treatments [(s,s) or (s,i)] can be performed per generation. The parameters used in the model are detailed in Table 2, with fitness cost values *w*_RR_ = 0.4 and *w*_RS_ = 0.2. Two levels of insecticide exposure *e(i)*, 20% and 50%, and two initial frequencies, corresponding to the presence of two [IF1; F(S) = 0.9977] or four [IF2; F(S) = 0.9955] resistant alleles (R) out of 880 analyzed, were considered.

**Appendix** **S1**: Supporting Information.
