# Supplementary figures and images for: Field detection and predicted evolution of spinosad resistance in Ceratitis capitata
Source: Pest Manag Sci. 2020 Jun 4;76(11):3702–10. doi: 10.1002/ps.5919 (PMC7587006; doi:10.1002/ps.5919)

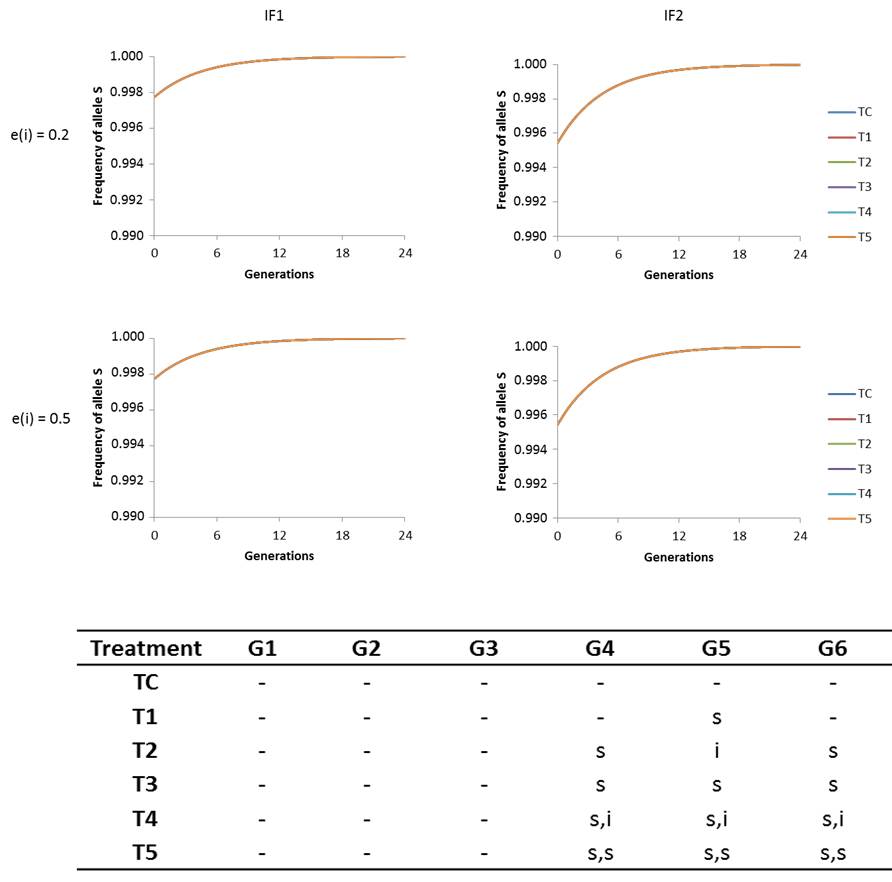

Supplement: Supplementary file 1 — Figure S1. Predicted rate of the evolution of the spinosad susceptible allele (S) frequency in field populations of Ceratitis capitata (24 generations, six generations (G1–G6)/year) when using spinosad (s) and a second insecticide without cross resistance (i), under different resistance management strategies (T1–T5 and TC). Note that one treatment [(s) or (i)] or two treatments [(s,s) or (s,i)] can be performed per generation. The parameters used in the model are detailed in Table 2, with fitness cost values w RR = 0.4 and w RS = 0.2. Two levels of insecticide exposure e(i), 20% and 50%, and two initial frequencies, corresponding to the presence of two [IF1; F(S) = 0.9977] or four [IF2; F(S) = 0.9955] resistant alleles (R) out of 880 analyzed, were considered. [file PS-76-3702-s001.jpg]
