# Supplementary material for: Field detection and predicted evolution of spinosad resistance in Ceratitis capitata
Source: Pest Manag Sci. 2020 Jun 4;76(11):3702–10. doi: 10.1002/ps.5919 (PMC7587006; doi:10.1002/ps.5919)
Supplement: Supplementary file 2 — Appendix S1: Supporting Information. [file PS-76-3702-s002.docx]

**Estimation of mutation rate for spinosad resistance**

Frequency of $S$ allele =$p$, frequency of $R$ allele =$q$, $p+q=1$

Selection coefficient $s$ for genotype $RR=s_{RR}$, selection coefficient for genotype $SR=s_{SR}$

$u$ stands for the mutation rate of wild type allele *S* to spinosad-resistant allele *R*

|  | **Genotypes** | | |
| --- | --- | --- | --- |
|  | $\boldsymbol{SS}$ | $\boldsymbol{SR}$ | $\boldsymbol{RR}$ |
| Zygote frequency  in generation *n* | $p_{n}^{2}$ | $2p_{n}q_{n}$ | $q_{n}^{2}$ |
| Selection coefficient  for each genotype | --- | $s_{SR}$ | $s_{RR}$ |
| Fitness of each genotype | $1$ | ${1-s}_{SR}$ | $1-s_{RR}$ |
| Contribution of each genotype to the next generation $n+1$ | $p_{n}^{2}$ | $2p_{n}q_{n}(1-s_{SR})$ | $q_{n}^{2}(1-s_{RR})$ |
| Relative frequency of each  genotype in generation $n+1$ | $\frac{p_{n}^{2}}{1-2p_{n}q_{n}s_{SR}-q_{n}^{2}s_{RR}}$ | $\frac{2p_{n}q_{n}(1-s_{SR})}{1-2p_{n}q_{n}s_{SR}-q_{n}^{2}s_{RR}}$ | $\frac{q_{n}^{2}(1-s_{RR})}{1-2p_{n}q_{n}s_{SR}-q_{n}^{2}s_{RR}}$ |

The frequency of $R$ $\mathrm{allele}$in generation $n+1$ is obtained by adding half the frequency of genotype *SR* and the frequency of genotype *RR* in generation $n+1$. Then,

$$q_{n+1}=\frac{p_{n}q_{n}\left( 1-s_{SR} \right)+q_{n}^{2}(1-s_{RR})}{1-2p_{n}q_{n}s_{SR}-q_{n}^{2}s_{RR}}$$

The change in the frequency of allele *R* from one generation to the next is

$$\Delta q=q_{n+1}-q_{n}=\frac{p_{n}q_{n}\left( 1-s_{SR} \right)+q_{n}^{2}\left( 1-s_{RR} \right)}{1-2p_{n}q_{n}s_{SR}-q_{n}^{2}s_{RR}}-q_{n}$$

$$\Delta q=\frac{-s_{SR}(p_{n}q_{n}+2p_{n}q_{n}^{2})-s_{RR}(q_{n}^{2}+q_{n}^{3})}{1-2p_{n}q_{n}s_{SR}-q_{n}^{2}s_{RR}}$$

Because the mutant allele *R* will be at low frequency (*q*) in the population, all the power terms of *q* can be discarded so that $\Delta q$ can be approximated by

$$\Delta q\approx\frac{-s_{SR}p_{n}q_{n}}{1-2p_{n}q_{n}s_{SR}}$$

**Selection-mutation equilibrium of the spinosad-resistant *R* allele.**

The mutation from *S* to *R* is considered but not the reciprocal mutation. Selection and mutation play opposite roles in the frequency of deleterious alleles in a population. There will be selection-mutation equilibrium when the net decrease of the resistant mutant allele *R* due to selection is compensated by the net increase in the amount of new *R* alleles generated by mutation; that is, when $\left| \Delta q \right|=\hat{u}p$ where *p* is the frequency of the wild type allele, and $\hat{u}$ is the estimated frequency of mutation from the wild type allele *S* to the mutant *R* allele. Thus,

$$\left| \frac{s_{SR}p_{n}q_{n}}{1-2p_{n}q_{n}s_{SR}} \right|=\hat{u}p_{n}$$

$$s_{SR}p_{n}q_{n}=\hat{u}p_{n}(1-2p_{n}q_{n}s_{SR})$$

$$\hat{u}=\frac{s_{SR}p_{n}q_{n}}{p_{n}(1-2p_{n}q_{n}s_{SR})}$$

eliminating $p_{n}$ from the numerator and denominator, replacing $p_{n} \mathrm{with} (1-q_{n})$ and eliminating the $q_{n}^{2}$ term in the denominator, it is obtained

$$\hat{u}\approx\frac{s_{SR}q_{n}}{1-2s_{SR}q_{n}}$$

**Conclusion.** For the population to attain the selection-mutation equilibrium regarding spinosad resistance, the estimated spinosad-resistant mutation rate ranges from $1,4\times{10}^{-5}$ to $8\times{10}^{-5}$ depending on the value of the selection coefficient and whether the spinosad-resistant males collected from the field are both heterozygous or homozygous for the resistant allele *R*.
